# Cross-Cultural Adaptation and Preliminary Psychometric Evaluation of the Romanian Version of the Revised MISSCARE Survey: A Single-Centre Pilot Study

**DOI:** 10.3390/healthcare14183122

**Published:** 2026-09-21

**Authors:** Claudia Raluca Balasa Virzob, Camelia Fizedean, Norberth-Istvan Varga, Adelina-Marioara Gherman, Mircea Iurciuc, Daian-Ionel Popa, Stela Iurciuc

**Affiliations:** 1 Department of Nursing, “Victor Babes” University of Medicine and Pharmacy, Eftimie Murgu Square, No. 2, 300041 Timisoara, Romania; virzob.claudia@umft.ro (C.R.B.V.); fizedean.camelia@umft.ro (C.F.); mavrea.adelina@umft.ro (A.-M.G.); iurciuc.mircea@umft.ro (M.I.); iurciuc.stela@umft.ro (S.I.); 2 Discipline of Medical Communications, Department II Microscopic Morphology, “Victor Babes” University of Medicine and Pharmacy, Eftimie Murgu Square No. 2, 300041 Timisoara, Romania; daian-ionel.popa@umft.ro

**Keywords:** missed nursing care, Revised MISSCARE Survey, nursing workforce, cross-cultural adaptation, psychometric evaluation, exploratory factor analysis

## Abstract

**Highlights:**

**What are the main findings?**

- The Romanian Revised MISSCARE Survey identified fundamental care, particularly ambulation and mobilization, as the most vulnerable area of nursing care.
- Exploratory analysis of Part B supported two preliminary factors with high internal consistency: organizational resources, coordination, and support; and staffing and workload pressure.

**What are the implications of the main findings?**

- Quality-improvement strategies should protect fundamental care while addressing support staffing, fluctuating patient demand, exhaustion, and workflow interruptions.
- The findings support progression to a national multicentre confirmatory study, but not yet national benchmarking or routine interhospital comparisons.

**Abstract:**

Background/Objectives: Missed nursing care is a patient-safety concern shaped by organizational resources, workload, and care prioritization. No Romanian version of the Revised MISSCARE Survey has previously undergone published cross-cultural and psychometric evaluation. This study translated and culturally adapted the instrument and examined its preliminary measurement properties and item-level findings in a Romanian hospital. Methods: A methodological, cross-sectional, single-centre pilot study invited all 487 nurses employed at a large university-affiliated hospital in western Romania. Translation and back-translation, expert review, and pretesting preceded electronic administration. Part A activities and Part B reasons were summarized descriptively. The Part B structure was investigated using polychoric correlations, parallel analysis, minimum-residual extraction, and promax rotation. McDonald’s omega and ordinal alpha assessed internal consistency. A unique-response-pattern sensitivity analysis evaluated stability. Results: Overall, 266 nurses participated (response proportion, 54.6%). Ambulation or mobilization was the most frequently missed activity, reported as missed at least occasionally by 55.3% of respondents, followed by bathing and skin care (36.8%), turning every two hours (36.5%), and mouth care (35.3%). The leading moderate or significant reasons were inadequate assistive or clerical personnel (84.6%), unexpected patient volume or acuity (83.5%), and inadequate staffing (82.7%). Parallel analysis supported two correlated Part B factors: organizational resources, coordination, and support; and staffing and workload pressure (r = 0.658). Omega was 0.968 and 0.943, respectively. The two-factor solution and item rankings remained stable in sensitivity analyses. Conclusions: The Romanian Revised MISSCARE Survey produced interpretable item-level findings and preliminary evidence of a two-factor structure for Part B, with high internal consistency within each factor.

## 1. Introduction

Patient safety is shaped not only by whether care is delivered correctly or incorrectly, but also by whether required care is delivered at all. In nursing, these omissions are captured by the concept of missed nursing care (MNC), defined as any aspect of required patient care that is omitted in part or in whole, or delayed [1,2]. Related literature has used the terms unfinished nursing care, care left undone, and implicitly rationed nursing care. Although these constructs arise from partly different theoretical traditions and are measured in different ways, they converge on a common outcome: necessary nursing work remains incomplete within the required time [3,4]. In this article, MNC is used as the overarching term because it corresponds to the construct and wording of the MISSCARE Survey.

MNC has been documented across countries and acute-care settings, although its measured frequency varies substantially with the instrument, clinical context, respondent population, recall period, and threshold used to define an omission. A recent systematic review and meta-analysis included 45 studies and 139,454 nurses; the pooled prevalence estimates for specific missed activities were 46% for ambulation, 36% for mouth care, 33% for emotional support, 31% for bathing, and 30% for feeding [5]. Heterogeneity exceeded 99% for these estimates, precluding interpretation as a single universal prevalence and emphasizing the importance of context-specific, item-level reporting. In the multicountry RN4CAST study, nurses in 12 European countries also reported care left undone, particularly time-intensive activities such as talking with and comforting patients and providing education [6]. Collectively, these findings position MNC as a recurrent international quality and safety signal rather than a problem confined to one specialty or health system.

The conditions associated with MNC are predominantly organizational. In a meta-analysis of 28 MISSCARE studies involving 20,768 nurses from 121 hospitals in 14 countries, the most frequently endorsed reasons were an unexpected increase in patient volume or acuity (78.1%), an inadequate number of staff (76.5%), and urgent patient situations (73.5%) [7]. A systematic review likewise found a consistent association between lower nurse staffing and omissions in nursing care [8]. The downstream evidence is primarily observational and should not be interpreted as proving causation; nevertheless, reviews have linked higher levels of unfinished or missed care with poorer perceived care quality and patient satisfaction, as well as medication errors, falls, urinary tract infections, pressure injuries, critical incidents, and readmissions, while evidence concerning mortality remains inconclusive [4,9]. At the workforce level, a meta-analysis of eight studies comprising 10,418 nurses found a significant negative association between MNC and job satisfaction (summary r = −0.294, 95% CI −0.364 to −0.219) [10].

Meaningful surveillance of MNC requires instruments that distinguish the nursing activities left incomplete from the circumstances perceived to contribute to those omissions. A systematic review of the psychometric literature identified three main instrument families—the MISSCARE Survey, the Basel Extent of Rationing of Nursing Care instrument, and the Tasks Undone scale—and found the MISSCARE Survey to be the most widely used tool validated across countries [11]. The MISSCARE Survey was developed as a two-part instrument measuring missed nursing care activities and reasons for missed care [12]. The version used as the basis for the 2019 revision comprised 24 Part A items and 17 Part B items [13]. The 2019 Revised MISSCARE Survey added adequate patient surveillance to Part A and five contemporary reasons to Part B, resulting in 25 activity items and 22 reason items [13]. In the US pilot involving 145 nursing staff, factor analysis was considered inappropriate for Part A because it represents distinct nursing actions, whereas exploratory analysis of Part B supported three broad domains concerning communication, labour resources, and material resources [13]. This two-part structure permits both item-level identification of care omissions and examination of potentially modifiable organizational reasons.

An instrument developed in one health system cannot be assumed to retain equivalent meaning or measurement properties after literal translation. Cross-cultural adaptation therefore requires systematic assessment of semantic, idiomatic, experiential, and conceptual equivalence, followed by psychometric re-evaluation in the target population [14]. Versions of the original MISSCARE Survey have been evaluated in several linguistic and organizational contexts, including Italian, Czech and Slovak, Danish, and Persian settings [15,16,17,18]. These studies support the feasibility of cross-cultural use but cannot substitute for evaluation of the expanded 2019 instrument. Direct cross-cultural evidence for the Revised MISSCARE Survey remains limited. A Norwegian pilot involving 120 nurses and nursing assistants identified four factors in Part B and recommended confirmation in larger and more diverse samples [19]. More recently, a Japanese study included 375 nurses with complete data and supported a three-factor Part B model, but several items were reallocated to reflect the Japanese context [20]. The difference between the US, Norwegian, and Japanese solutions indicates that the organization of perceived reasons for MNC may be context-dependent and should be tested rather than presumed.

Important gaps consequently remain. To our knowledge, no Romanian-language version of the Revised MISSCARE Survey has undergone published cross-cultural adaptation and psychometric evaluation. Although pilot evidence based on the original survey is available from the Czech Republic and Slovakia [16], the absence of an evaluated Romanian instrument limits standardized assessment within Romania and comparability with international studies. The gap is not solely linguistic: the Revised MISSCARE Survey itself has comparatively limited independent cross-cultural evidence, and the available studies have not reproduced an identical Part B structure [13,19,20]. A transparent Romanian pilot is therefore required to establish conceptual suitability, characterize item performance, and generate a candidate measurement model before attempting national estimates or confirmatory testing. Such a staged approach also avoids presenting evidence from one institution as definitive validation for the wider Romanian nursing workforce.

Accordingly, the primary aim of this study was to translate and cross-culturally adapt the Revised MISSCARE Survey for Romanian-speaking nurses and to conduct a preliminary psychometric evaluation of the resulting Romanian version in a large university-affiliated hospital. The secondary aims were to describe, at item level, the reported frequency of missed nursing care activities and the perceived reasons for missed care, and to explore the factor structure and internal consistency of Part B. The study was explicitly framed as a single-centre pilot intended to provide a reproducible foundation and a candidate factor structure for subsequent independent confirmatory factor analysis and broader multicentre evaluation across Romania, rather than to establish nationally representative estimates or final national validation.

## 2. Materials and Methods

### 2.1. Study Design

This was a methodological, cross-sectional, single-centre pilot study using a single anonymous electronic administration; no intervention, follow-up, or repeated measurement was undertaken.

The study comprised two linked phases: (1) translation, cross-cultural adaptation, expert review, and pretesting of the Romanian version; and (2) hospital-wide field administration followed by item-level descriptive and exploratory psychometric analyses. Given the exploratory nature of this first single-centre evaluation, confirmatory factor analysis, test–retest reliability, responsiveness, and criterion or convergent validity against an external instrument were outside the scope of the pilot and were reserved for subsequent independent multicentre evaluation.

In measurement-property terms, this pilot evaluated the exploratory structural validity and internal consistency of Part B. It did not evaluate cross-cultural validity or measurement invariance between the Romanian and source-language versions.

Reporting was guided by the Strengthening the Reporting of Observational Studies in Epidemiology statement for cross-sectional studies, the Consensus-Based Checklist for Reporting of Survey Studies, and the applicable items of the Checklist for Reporting Results of Internet E-Surveys [21,22,23].

### 2.2. Setting

The study was conducted at the Clinical Municipal Emergency Hospital of Timișoara (Spitalul Clinic Municipal de Urgență Timișoara), a large public university-affiliated hospital in Timișoara, western Romania. The institution provides medical, surgical, emergency, critical-care, and other specialist services across multiple clinical locations and organizational units. Field administration took place in August 2026.

For manuscript reporting, clinical units were not identified individually. Before analysis, named units were mapped to three investigator-validated service groups: Medical, Surgical, and Other. Medical comprised non-operative medical specialties; Surgical comprised operative and surgical specialties; and Other comprised emergency, critical-care, and cross-cutting clinical services not appropriately classified in the first two groups. These broad groups were used only for aggregate sample description. No unit-level ranking, unit-specific response reporting, department-by-demographic cross-tabulation, or inferential comparison among the three service groups was planned.

### 2.3. Participants, Sampling, and Recruitment

The target population comprised nurses employed by the participating hospital. A census rather than a sampled recruitment strategy was used: the electronic invitation was distributed to all 487 nurses in the hospital-wide sampling frame, without restriction by department, clinical specialty, work schedule, educational route, or length of professional experience. Employment as a nurse at the participating institution during the recruitment period was the eligibility criterion; no minimum period of employment was required.

Because the complete finite target population was invited, no a priori hypothesis-testing power calculation or probability-based sample-size calculation was used. The recruitment objective was to obtain the largest feasible pilot dataset from the available population. Adequacy for exploratory factor analysis was evaluated empirically using the participant-to-item ratio together with the Kaiser–Meyer–Olkin index, Bartlett’s test of sphericity, the correlation structure, and the stability analyses specified below.

The study invitation and survey link were relayed electronically through the hospital’s nursing communication channels, including the head nurses of clinical units. The invitation explained the study purpose, voluntary nature of participation, anonymous handling of responses, and absence of employment-related consequences for participation or non-participation. No financial or other incentive was offered. Investigators instructed nurses to complete the questionnaire individually, to answer from their own perspective and experience, and to submit one questionnaire per participant.

### 2.4. Instrument

The study used the 2019 Revised MISSCARE Survey described by Dabney, Kalisch, and Clark (2019) [13], which builds on the original MISSCARE Survey developed by Kalisch and Williams (2009) [12]. Written authorization to translate the revised instrument into Romanian was obtained from Beverly W. Dabney, PhD, RN, before field administration.

The administered questionnaire contained 69 variables: 22 demographic and work-context variables, 25 Part A items, and 22 Part B items. The contextual variables covered the participant’s unit, education and nursing qualification, sex, age, professional role, usual working hours and schedule, experience, recent overtime and absence, intention to leave, perceived staffing adequacy, workload indicators, and satisfaction with the current position, nursing profession, and teamwork.

Part A asks respondents how frequently specified nursing care activities are missed by nursing staff on their unit, including the respondent. The 25 activities are rated on a five-category ordinal scale and were coded as 1 = never missed, 2 = rarely missed, 3 = occasionally missed, 4 = frequently missed, and 5 = always missed. Higher values therefore indicate more frequently missed care. Part B contains 22 potential reasons for missed nursing care and uses a four-category ordinal scale coded as 1 = not a reason, 2 = minor reason, 3 = moderate reason, and 4 = significant reason. Higher values indicate greater perceived importance of the reason. No item was reverse-scored.

Part A was treated as a formative inventory of clinically distinct activities rather than as a reflective unidimensional scale. Consequently, no total Part A score was designated as a primary endpoint and no factor analysis of Part A was planned. Part B was treated as potentially multidimensional and was evaluated using exploratory factor analysis. The full response distribution for every Part A and Part B item was retained for reporting rather than reducing the instrument to a single summary score.

### 2.5. Translation and Cross-Cultural Adaptation

Translation and cross-cultural adaptation followed the staged principles proposed by Beaton et al. (2000) [14], with emphasis on semantic, idiomatic, experiential, and conceptual equivalence rather than literal word-for-word translation.

First, two professional translators independently translated the English-language Revised MISSCARE Survey into Romanian. Both translators were native Romanian speakers with high proficiency in English. Neither had previous familiarity with the MISSCARE Survey or its underlying construct. The translators and research team compared the two forward translations item by item and reconciled them by consensus into one Romanian version, considering the questionnaire instructions, response options, clinical terminology, conceptual meaning, and natural Romanian usage.

Second, two additional professional translators independently translated the reconciled Romanian version back into English. Both were native Romanian speakers with high proficiency in English, had not participated in the forward translation, were unfamiliar with the MISSCARE Survey and its construct, and were blinded to the wording of the original instrument. The research team compared both back-translations with the English source instrument, documented and discussed discrepancies, and revised the Romanian wording until conceptual equivalence was judged satisfactory. The instrument developer authorized the Romanian translation but did not review the final back-translated version.

Third, an expert committee reviewed the source instrument, forward translations, reconciled version, back-translations, and documented discrepancies. The committee comprised three assistant professors in nursing who were also registered nurses with clinical, educational, and research experience. The review addressed semantic, idiomatic, experiential, and conceptual equivalence, as well as clarity, relevance, and applicability within Romanian nursing practice. Decisions were reached by consensus. No item was deleted, added, or assigned a different response structure during this process.

Finally, the pre-final version was pretested individually with 20 head nurses representing different clinical areas. This was a structured review of clarity, comprehensibility, cultural appropriateness, and response options rather than a formal cognitive-interviewing procedure; paraphrasing and probing techniques were not used. All 20 participants reported that they understood the questions and were able to provide clear and specific responses. No item was identified as incomprehensible or culturally inapplicable. Minor revisions were limited to linguistic clarity and natural Romanian usage and did not change item content or response structure. Data collected during pretesting were not merged with the main field-administration database.

The resulting Romanian-language version of the Revised MISSCARE Survey was used in the hospital-wide pilot and is provided in the Supplementary File.

### 2.6. Electronic Survey Administration

The Romanian questionnaire was implemented as a self-administered electronic survey in Google Forms. The landing information described the study, the voluntary nature of participation, confidentiality protections, and the instruction to complete the survey individually. Respondents accessed the form using a personal or workplace electronic device at a time available to them during the data-collection window. All questionnaire items displayed in the study form required a response before submission.

The survey did not request names, email addresses, employee identification numbers, contact details, or other direct personal identifiers. Authentication was required solely to enable the Google Forms setting limiting each account to one submission; authentication details were not collected with the questionnaire responses or included in the analytical dataset. Submission timestamps were retained, but device identifiers and Internet Protocol addresses were not collected. Participation was uncompensated. Submission of the completed anonymous questionnaire constituted implied informed consent. A respondent could leave the form before submission without transmitting a response; because submitted records were anonymous, an individual record could not subsequently be linked back to a participant for withdrawal.

After field administration had been completed, the research team learned that some nurses had completed the questionnaire while working alongside groups of approximately two to five colleagues. Although the instructions requested individual completion, the affected submissions could not be identified retrospectively from the available metadata. This issue was therefore treated as a potential source of dependence and examined through the response-pattern sensitivity analysis described below.

### 2.7. Data Management, Coding, and Confidentiality

Responses were exported from Google Forms to a Microsoft Excel workbook. The final source workbook was frozen, retained unchanged, and treated as read-only. All coding, recoding, and derived variables were created in a separate analytical dataset. A codebook and an auditable cleaning log documented source columns, Romanian item wording, English reporting labels, scoring, and every transformation.

Three non-substantive response-label variants were harmonized before scoring: the Romanian label equivalent to “always” without the qualifier “missed” was coded as always missed; a grammatical variant of “frequently missed” was coded as frequently missed; and a typographical variant of “significant reason” was coded as significant reason. These operations changed labels only and did not alter the intended response category. No statistical imputation was used. Descriptive denominators were to be stated item by item, and exploratory factor analysis required complete data across all Part B items.

The three open-response workload fields concerning numbers of patients, admissions, and discharges were not included in quantitative synthesis because the electronic form did not enforce a single unit or reference period for those entries. Named-unit values were retained only in a restricted mapping file and were replaced in manuscript-facing outputs by Medical, Surgical, or Other. Access to individual-level data and the restricted unit mapping was limited to the research team. Public reporting was designed to avoid small-cell disclosure and deductive identification; raw unit names, unit-specific counts, and unit-by-demographic combinations were not reported.

### 2.8. Ethical Considerations

The study was conducted in accordance with the Declaration of Helsinki, applicable Romanian requirements, and Regulation (EU) 2016/679 on the protection of personal data. Approval was granted by the Ethics Committee of the Clinical Municipal Emergency Hospital of Timișoara before data collection (approval no. E-2857, 16 June 2026).

Potential participants received written information about the study purpose, procedures, voluntary participation, confidentiality, and their right to decline or discontinue completion before submission without penalty. Participation or non-participation had no intended effect on employment, professional evaluation, or the relationship with hospital management. No directly identifying information was collected, and analyses and reporting used aggregate data only.

### 2.9. Statistical Analysis

The statistical analysis plan was finalized and locked on 17 August 2026 after data collection and before substantive interpretation of the psychometric findings. Any analysis added after this lock was to be identified as exploratory or post hoc. The primary analysis retained every submitted questionnaire meeting the study definition; records were not removed solely because a complete response vector exactly matched another submitted vector.

A robustness analysis was defined using exact equality across the complete 69-variable response vector. For this sensitivity dataset, response vectors were assigned deterministic pattern identifiers and the first record for each distinct complete pattern was retained. The sensitivity analysis did not replace the primary analysis and was not interpreted as establishing which submissions originated from distinct individuals.

Categorical participant and work-context variables were summarized using counts and percentages. Ordinal item responses were summarized using the complete category distribution, mean and sample standard deviation for comparability with previous MISSCARE literature, and median and interquartile range as distribution-sensitive diagnostics. Percentages for Part A additionally summarized the proportion scored 3–5 (at least occasionally missed), whereas Part B percentages summarized the proportion scored 3–4 (a moderate or significant reason). Items were ranked descriptively by these prespecified threshold proportions. No non-response weighting was applied because individual characteristics of nonrespondents were unavailable.

Part A results were analyzed item by item. No overall Part A score, inferential comparison among Medical, Surgical, and Other, or department-level ranking was planned. Statistical testing was not used to compare the primary and sensitivity datasets; emphasis was placed on the magnitude and concordance of descriptive and psychometric estimates.

Structural validity was explored for the 22 ordinal Part B items. Pairwise polychoric correlations were estimated by maximum likelihood from the observed ordinal contingency tables. The raw polychoric correlation matrix was inspected for positive definiteness. If it was non-positive-definite, a spectral eigenvalue-clipping correction was applied: eigenvalues below 10^−6^ were replaced by 10^−6^, the matrix was reconstructed, and the resulting matrix was rescaled to a unit diagonal before factor extraction. The need for this correction and the properties of the raw matrix were reported transparently.

Sampling adequacy was evaluated using the overall and item-level Kaiser–Meyer–Olkin indices and Bartlett’s test of sphericity, calculated from the conventional Pearson item-correlation matrix and explicitly labelled as such. Factor retention was determined using parallel analysis based on 2000 normally distributed random datasets, with observed eigenvalues compared against the 95th percentile of random eigenvalues, together with the scree plot and clinical interpretability.

Factors were extracted using minimum-residual estimation and rotated using oblique promax rotation, because correlated dimensions were expected. Interpretation was based primarily on the pattern matrix while also examining structure coefficients, communalities, uniquenesses, and factor correlations. Absolute pattern coefficients of at least 0.40 were considered salient. Cross-loadings were examined at an absolute coefficient of at least 0.30; a difference of less than 0.20 between the two largest absolute pattern coefficients was considered potentially ambiguous. Model residual fit was summarized using the root mean square residual. Factor labels were assigned only after joint statistical and clinical review of the contributing items. Confirmatory factor analysis was not fitted in this pilot dataset to avoid deriving and confirming a model in the same single-centre sample.

Internal consistency was estimated separately for each interpretable Part B factor. McDonald’s omega, estimated separately for the items assigned to each retained factor, was the primary internal-consistency coefficient, and ordinal Cronbach’s alpha, calculated from the relevant polychoric submatrix, was secondary. Ninety-five percent percentile confidence intervals were estimated using 500 bootstrap replicates. Bootstrap sampling used the exact complete response-pattern identifier as the resampling unit, thereby preserving all rows belonging to a sampled pattern.

Primary and unique-pattern sensitivity analyses were compared using differences in item means and prespecified threshold proportions, Spearman correlations between item rankings, the number of factors supported by parallel analysis, factor pattern coefficients, root mean square residual, factor-specific internal-consistency coefficients, and Tucker’s coefficient of congruence after factor alignment. Congruence was calculated after matching factors by the maximum absolute similarity and aligning their signs. No null-hypothesis significance test was applied to differences between the two analytical datasets.

Analyses were implemented in Python 3.12.13 using NumPy 2.3.5, pandas 2.2.3, and SciPy 1.17.0, together with purpose-written analysis functions for polychoric correlations, minimum-residual extraction, promax rotation, ordinal reliability, parallel analysis, and factor-solution alignment. The random-number seed was fixed at 20,260,817. The frozen-source checksum, executable analysis code, codebook, cleaning log, aggregated output tables, software versions, and random seed were retained as the reproducibility record. Individual-level records and raw named-unit mappings were excluded from the public reproducibility package because of deductive-disclosure risk.

## 3. Results

### 3.1. Participants and Response Rate

Of the 487 nurses invited to participate, 266 submitted a complete questionnaire, corresponding to a response proportion of 54.6%. All 266 questionnaires contained complete responses for the 69 variables included in the analytical dataset; therefore, no record was excluded and no imputation was required. Table 1 presents the participant and work-context characteristics.

Participants worked in medical departments (n = 137, 51.5%), surgical departments (n = 96, 36.1%), or other departments (n = 33, 12.4%). Most participants were women (n = 227, 85.3%), and the largest age group was 45–54 years (n = 122, 45.9%). A bachelor’s degree was the most frequently reported general educational level (n = 130, 48.9%), whereas post-secondary nursing school was the most frequently reported nursing qualification (n = 115, 43.2%).

Rotating day/evening/night shifts were reported by 142 participants (53.4%). More than 10 years of experience were reported by 176 participants (66.2%) in their current role and by 159 (59.8%) on their current unit. Staffing was perceived as adequate no more than 25% of the time by 122 participants (45.9%), whereas 11 (4.1%) reported adequate staffing 100% of the time. Overall, 156 participants (58.6%) were satisfied or very satisfied with their current position, 197 (74.1%) with the nursing profession, and 187 (70.3%) with teamwork.

### 3.2. Missed Nursing Care Activities

The proportion of participants reporting that an activity was missed at least occasionally ranged from 6.4% to 55.3%. The most frequently missed activity was ambulation or mobilization three times daily or as ordered (n = 147, 55.3%; mean 3.01, SD 1.54). This was followed by patient bathing and skin care (36.8%), turning the patient every 2 h (36.5%), mouth care (35.3%), and feeding the patient while food was still warm (28.6%).

**Figure 1:**
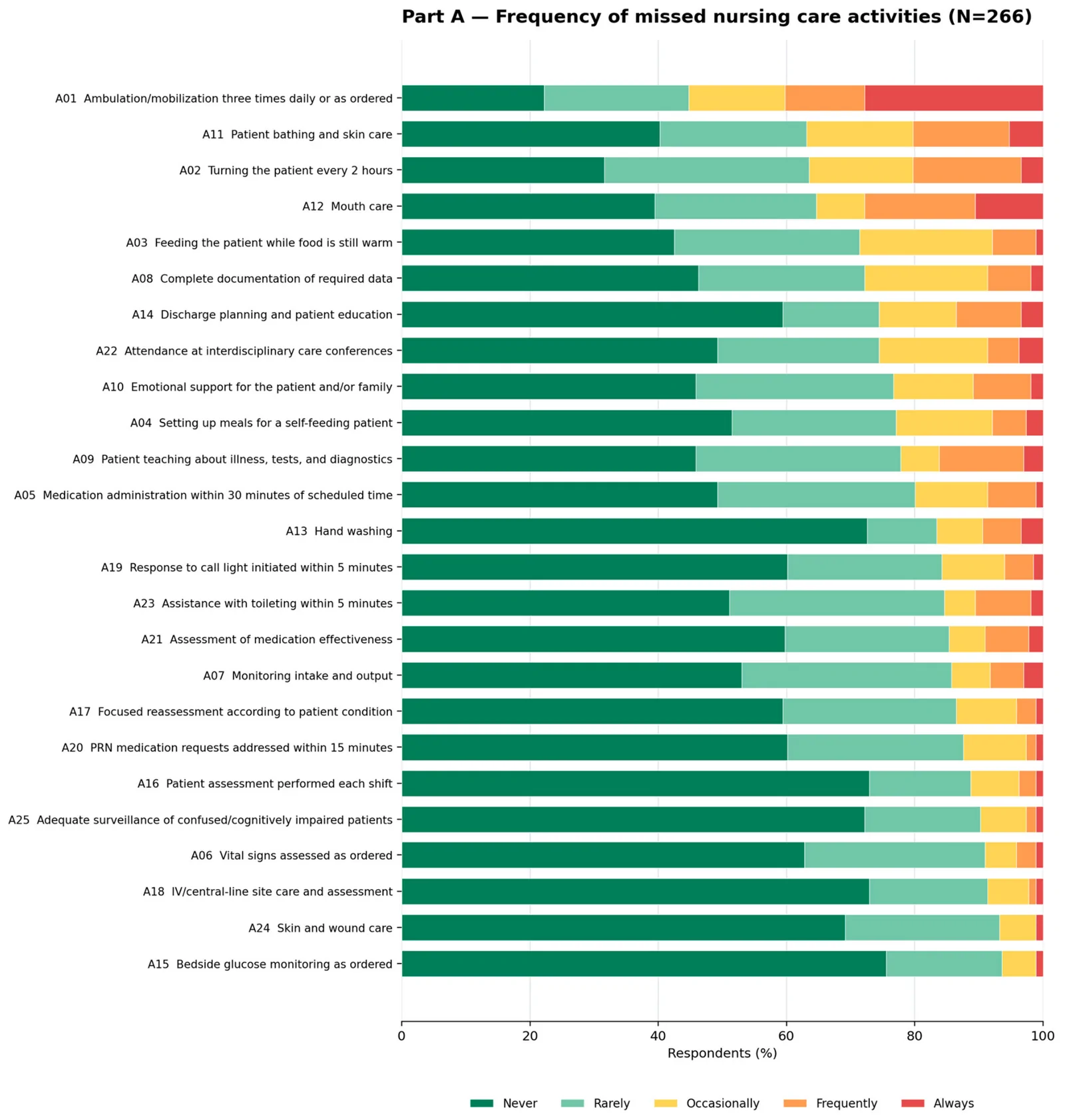
Distribution of Responses to Part A Items, Ordered by the Proportion Missed at Least Occasionally.

The lowest proportions were observed for bedside glucose monitoring as ordered (6.4%), skin and wound care (6.8%), intravenous/central-line site care and assessment (8.6%), vital signs assessed as ordered (9.0%), and adequate surveillance of confused or cognitively impaired patients (9.8%). Item-level descriptive statistics and response distributions are presented in Table 2, and the complete distribution across the five response categories is shown in Figure 1.

### 3.3. Reasons for Missed Nursing Care

The proportion rating a reason as moderate or significant ranged from 41.4% to 84.6%. The most frequently endorsed reason was inadequate assistive and/or clerical personnel (n = 225, 84.6%; mean 3.41, SD 0.91). Other highly endorsed reasons were an unexpected increase in patient volume and/or acuity (83.5%), an inadequate number of staff (82.7%), emotional or physical exhaustion (77.4%), and interruptions and simultaneous tasks (77.1%).

**Figure 2:**
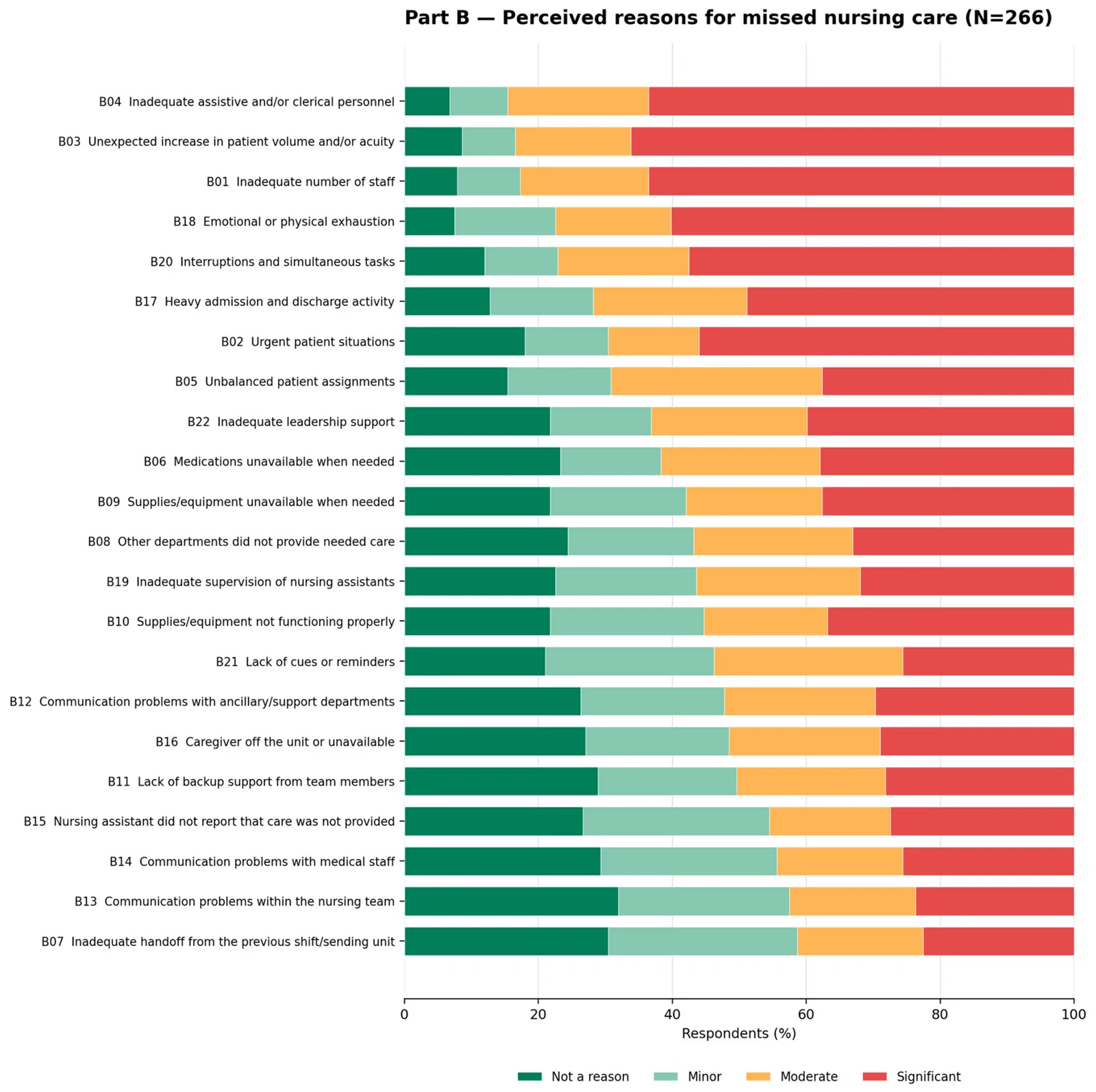
Distribution of Responses to Part B Items, Ordered by the Proportion Rated a Moderate or Significant Reason.

The lowest proportions were recorded for inadequate handoff from the previous shift or sending unit (41.4%), communication problems within the nursing team (42.5%), communication problems with medical staff (44.4%), failure of a nursing assistant to report that care had not been provided (45.5%), and lack of backup support from team members (50.4%). Table 3 presents the item-level results, while Figure 2 shows the distribution across all four response categories.

### 3.4. Exploratory Factor Structure of Part B

The 22 Part B items were suitable for exploratory analysis. The Kaiser–Meyer–Olkin index, calculated from the Pearson item-correlation matrix, was 0.908, and Bartlett’s test of sphericity was statistically significant, χ^2^ (231) = 4997.17, *p* < 0.001. The raw pairwise polychoric correlation matrix was slightly non-positive-definite (minimum eigenvalue −0.033; two negative eigenvalues) and was smoothed to a positive-definite correlation matrix before extraction.

Parallel analysis based on 2000 random datasets supported two factors. The first two observed eigenvalues (13.452 and 2.460) exceeded their corresponding 95th-percentile random eigenvalues, whereas the third observed eigenvalue (1.177) was below the random 95th percentile (1.444). The two-factor minimum-residual solution with promax rotation had an RMSR of 0.061.

Factor 1 comprised 14 items (B06–B16, B19, B21, and B22), with primary pattern coefficients ranging from 0.624 to 0.962, and was provisionally labelled “Organizational resources, coordination, and support.” Factor 2 comprised eight items (B01–B05, B17, B18, and B20), with primary pattern coefficients ranging from 0.591 to 1.093, and was provisionally labelled “Staffing and workload pressure.” The factors were positively correlated (r = 0.658). Complete pattern and structure coefficients, communalities, uniqueness values, and item assignments are presented in Table 4. The pattern coefficient of 1.093 for B01 was verified. In the oblique solution, this value resulted from the combination of correlated factors and a negative cross-loading on Factor 1 rather than from a communality estimate exceeding 1.0.

### 3.5. Internal Consistency

Both retained factors showed high internal consistency (Table 5). For Factor 1, McDonald’s omega was 0.968 (95% CI 0.956–0.976) and ordinal alpha was 0.967 (95% CI 0.955–0.976). For Factor 2, omega was 0.943 (95% CI 0.919–0.961) and ordinal alpha was 0.943 (95% CI 0.917–0.961).

### 3.6. Sensitivity Analysis

The sensitivity analysis retained one observation for each exact complete 69-variable response pattern (n = 200). Results were materially unchanged (Table 6). The maximum absolute difference from the primary analysis was 3.5 percentage points for Part A threshold proportions and 3.6 percentage points for Part B threshold proportions. Item rankings remained highly concordant (Spearman ρ = 0.993 for Part A and ρ = 0.995 for Part B).

Parallel analysis again retained two factors, and the sensitivity solution had an RMSR of 0.062. Tucker congruence coefficients comparing the primary and sensitivity factor solutions were 0.995 for Factor 1 and 0.990 for Factor 2.

## 4. Discussion

### 4.1. Principal Findings

To our knowledge, this study provides the first preliminary psychometric evaluation of the Romanian Revised MISSCARE Survey. The Romanian version produced clinically interpretable item-level findings and an internally consistent two-factor solution for Part B, with closely concordant results in the unique-response-pattern sensitivity analysis. The response proportion of 54.6% supports the feasibility of hospital-wide electronic administration but does not establish representativeness beyond the participating hospital or definitive national validation.

Because recruitment and eligibility differed across adaptation studies, the present findings should be regarded as preliminary evidence supporting further evaluation rather than definitive national validation [16,17,19].

### 4.2. Patterns of Missed Care and Contributing Reasons

Ambulation or mobilization was the most frequently missed activity, with 55.3% of respondents reporting it as missed at least occasionally and a mean score of 3.01. This is consistent with its first-place ranking in the US Revised MISSCARE pilot (mean 3.13) and the Czech and Slovak samples, and with the 74.8% reported in the Italian validation study [13,15,16]. The pooled estimate for ambulation was 46% in a recent meta-analysis, while pooled estimates for mouth care, bathing, and feeding were 36%, 31%, and 30%, respectively [5]. The corresponding Romanian proportions for mouth care (35.3%), bathing and skin care (36.8%), and feeding while food was warm (28.6%) were descriptively similar. These comparisons must remain contextual because the meta-analysis reported very high between-study heterogeneity and included differences in setting, instrument version, recall frame, and reporting threshold [5].

By contrast, bedside glucose monitoring, skin and wound care, intravenous or central-line assessment, and vital-sign assessment were among the least frequently missed activities. A similar ordering was observed in the US Revised MISSCARE pilot, in which glucose monitoring, intravenous-line care, and vital-sign assessment were also near the lower end of Part A [13]. Taken together, the pattern suggests that, when work is constrained, repetitive fundamental activities may be more vulnerable than scheduled technical or monitoring tasks. This should be interpreted as possible implicit prioritization under workload pressure, not as evidence of individual negligence. Studies using other unfinished-care measures have likewise linked time pressure and lower staffing with care left undone, but their estimates are not directly interchangeable with MISSCARE results [3,8,24].

The dominant perceived reasons in Part B were inadequate assistive or clerical personnel (84.6%), unexpected patient volume or acuity (83.5%), and inadequate staffing (82.7%). This pattern was internally consistent with 45.9% of respondents reporting adequate staffing no more than 25% of the time. The latter two correspond closely to the leading pooled reasons in the international MISSCARE meta-analysis: unexpected volume or acuity (78.1%) and inadequate staff (76.5%) [7]. The Italian validation study similarly reported unexpected patient increases (90.5%) and insufficient human resources (85.5%) among its leading reasons [15]. The particularly high Romanian endorsement of inadequate assistive or clerical personnel may reflect local task distribution and limited support capacity, but the present design cannot identify the responsible mechanism. Emotional or physical exhaustion (77.4%) and interruptions or simultaneous tasks (77.1%) further indicate that perceived workload pressure extends beyond headcount. Although communication-related reasons ranked lower, they were still endorsed as moderate or significant by approximately 41–50% of respondents and should not be considered negligible.

### 4.3. Psychometric Interpretation

Part A was intentionally analysed as an inventory of clinically distinct nursing activities rather than as a reflective scale. This is consistent with the Revised MISSCARE pilot, which considered factor analysis inappropriate for Part A because its items represent independent nursing actions [13]. The absence of a total Part A construct should therefore not be interpreted as a psychometric deficiency; item-level distributions and rankings are the clinically relevant outputs.

For Part B, the two-factor solution is plausible and not without precedent. In the Czech and Slovak versions, the original 17-item Part B also separated into two components, combining communication with material resources in one component and labour resources in the other [16]. The Romanian first factor similarly brought together resources, communication, coordination, and organizational support, whereas the second concentrated on staffing, workload, admissions and discharges, exhaustion, and interruptions. However, the original and Revised US studies identified three domains, while four factors were reported in Italy and Norway, five in the Persian version, and three in the Danish and Japanese studies [12,13,15,17,18,19,20]. These differences likely reflect both healthcare context and analytical choices, including instrument version, correlation matrix, extraction method, rotation, and exploratory versus confirmatory modelling. The Romanian labels are therefore provisional. The factor correlation of 0.658 indicates related organizational dimensions rather than orthogonal processes and supports oblique rotation.

The high internal-consistency estimates for both Romanian factors were compatible with those reported across MISSCARE adaptations, although previous studies differed in whether reliability was reported for Part B overall or for individual factors [13,15,16,17,18,19,20]. However, coefficients above 0.90 do not demonstrate validity or unidimensionality and may also reflect overlapping item content [25]. Independent confirmation is therefore more important than further maximizing reliability.

### 4.4. Future Research Directions, Strengths and Limitations

The findings identify system-level priorities rather than individual or unit-level performance failures. Potential responses include reviewing the availability and deployment of assistive personnel, staffing relative to patient acuity, admission and discharge workload, task interruptions, and workflows that protect ambulation, hygiene, repositioning, oral care, and feeding. MISSCARE results should be used for service improvement and repeated monitoring, not punitive comparisons between small clinical units. Because patient outcomes were not measured, the present study cannot establish that the reported omissions caused adverse events, despite such associations being described elsewhere [4,8,9].

The next stage should be a multicentre Romanian study including different regions, hospital types, and service profiles. The present two-factor solution should be specified prospectively and tested by confirmatory factor analysis in an independent sample. Further evaluation should include test–retest reliability, convergent or criterion-related validity, and, where sample size permits, measurement invariance across medical, surgical, and other services and across hospital groups. This progression is consistent with cross-cultural measurement principles and with recommendations from previous MISSCARE adaptation studies [11,14,19,20].

Strengths include authorization from the Revised MISSCARE Survey author, rigorous translation and back-translation, expert and respondent review, census invitation of all 487 hospital nurses, complete item-level reporting, and analysis methods suited to ordinal data. Factor retention used parallel analysis rather than the eigenvalue-greater-than-one rule alone, internal consistency was assessed using both omega and ordinal alpha with bootstrap confidence intervals, and a sensitivity analysis evaluated the influence of repeated exact response patterns. Reporting departments only as Medical, Surgical, and Other protected confidentiality in a setting where finer demographic cross-tabulation could identify individual respondents.

Adaptation-related limitations should first be considered. Pretesting involved head nurses rather than a broader sample of bedside nurses and used structured individual feedback rather than formal cognitive interviewing. Although all pretest participants reported understanding the questions and being able to answer them clearly, comprehensibility across the entire intended population of hospital nurses was not established.

This was a cross-sectional study in one university-affiliated hospital, and the results cannot be generalized to all Romanian nurses or hospitals. Nonresponse bias is possible because 45.4% of invited nurses did not participate and no information was available to compare respondents with nonrespondents. MISSCARE captures staff perceptions rather than directly observed care events, and no patient outcomes were collected; causal or outcome-related conclusions are therefore unwarranted. Mandatory electronic fields produced complete data but do not by themselves establish item acceptability. The study did not include test–retest assessment or an independent confirmatory sample. Aggregating departments protected participants but prevented evaluation of potentially important unit-level variation.

The slightly non-positive-definite polychoric correlation matrix required a spectral eigenvalue correction before factor extraction. Because modification of a correlation matrix may affect eigenvalues and loading estimates, the resulting two-factor solution remains provisional and requires independent confirmation.

After data collection, the investigators learned that some nurses had completed the questionnaire alongside groups of approximately two to five colleagues while on duty. Although the instructions envisaged individual completion, collaborative responding may have reduced independence, encouraged response convergence, narrowed variability, or inflated associations and reliability. All 266 submitted questionnaires were retained because they constituted the confirmed final dataset and individual participation in group completion could not be reconstructed. The close agreement between the primary and unique-response-pattern sensitivity analyses reduces concern that exact duplicate response patterns determined the findings; however, it cannot restore independence or eliminate possible group-response bias.

## 5. Conclusions

The Romanian Revised MISSCARE Survey was feasible for hospital-wide administration and produced interpretable item-level findings and preliminary evidence of a two-factor structure for Part B, with high internal consistency within each factor. Fundamental nursing activities were more frequently reported as missed, while staffing, assistive resources, workload, exhaustion, and interruptions dominated the perceived reasons. The two-factor solution and high internal consistency support continued evaluation, but they do not constitute definitive national validation. A multicentre Romanian study with independent confirmatory testing and additional reliability and validity evidence is required before national benchmarking or routine interhospital comparisons.

## Figures and Tables

**Table 1:** Participant and Work-Context Characteristics (n = 266).

Characteristic	Category	n (%)
Department group	Medical	137 (51.5)
	Surgical	96 (36.1)
	Other	33 (12.4)
Sex	Female	227 (85.3)
	Male	39 (14.7)
Age group	<35 years	41 (15.4)
	35–44 years	49 (18.4)
	45–54 years	122 (45.9)
	55–64 years	54 (20.3)
Highest education	Bachelor’s degree	130 (48.9)
	Short-cycle higher education	66 (24.8)
	High school or equivalent	38 (14.3)
	Postgraduate degree	32 (12.0)
Highest nursing qualification	Post-secondary nursing school	115 (43.2)
	University nursing degree	97 (36.5)
	Master’s degree in a medical field	31 (11.7)
	Other qualification	23 (8.6)
Work schedule	Rotating day/evening/night shifts	142 (53.4)
	Day shift	104 (39.1)
	Night shift	20 (7.5)
Experience in current role	>10 years	176 (66.2)
	>5–10 years	51 (19.2)
	>2–5 years	28 (10.5)
	>6 months–2 years	11 (4.1)
Experience on current unit	>10 years	159 (59.8)
	>5–10 years	62 (23.3)
	>2–5 years	27 (10.2)
	>6 months–2 years	13 (4.9)
	≤6 months	5 (1.9)
Works mainly in the stated unit	Yes	259 (97.4)
	No	7 (2.6)
Usual weekly working hours	≥30 h	259 (97.4)
	<30 h	7 (2.6)
Overtime in previous 3 months	None	115 (43.2)
	>12 h	90 (33.8)
	1–12 h	61 (22.9)
Sickness/other absence in previous 3 months	None	246 (92.5)
	At least one day/shift	20 (7.5)
Intention to leave current position	No intention to leave	239 (89.8)
	Within 12 months	27 (10.2)
Perceived staffing adequacy	0% of the time	56 (21.1)
	25% of the time	66 (24.8)
	50% of the time	72 (27.1)
	75% of the time	61 (22.9)
	100% of the time	11 (4.1)
Satisfaction with current position	Dissatisfied/very dissatisfied	59 (22.2)
	Neutral	51 (19.2)
	Satisfied/very satisfied	156 (58.6)
Satisfaction with the nursing profession	Dissatisfied	29 (10.9)
	Neutral	40 (15.0)
	Satisfied/very satisfied	197 (74.1)
Satisfaction with teamwork	Dissatisfied/very dissatisfied	29 (10.9)
	Neutral	50 (18.8)
	Satisfied/very satisfied	187 (70.3)

**Table 2:** Distribution of Responses to Part A: Missed Nursing Care Activities (N = 266).

Item/Activity	Mean (SD)	Never %	Rarely %	Occasionally %	Frequently %	Always %	≥Occasionally %
A01 Ambulation/mobilization three times daily or as ordered	3.01 (1.54)	22.2	22.6	15.0	12.4	27.8	55.3
A02 Turning the patient every 2 h	2.29 (1.18)	31.6	32.0	16.2	16.9	3.4	36.5
A03 Feeding the patient while food is still warm	1.95 (1.00)	42.5	28.9	20.7	6.8	1.1	28.6
A04 Setting up meals for a self-feeding patient	1.82 (1.04)	51.5	25.6	15.0	5.3	2.6	22.9
A05 Medication administration within 30 min of scheduled time	1.80 (0.99)	49.2	30.8	11.3	7.5	1.1	19.9
A06 Vital signs assessed as ordered	1.52 (0.82)	62.8	28.2	4.9	3.0	1.1	9.0
A07 Monitoring intake and output	1.73 (1.00)	53.0	32.7	6.0	5.3	3.0	14.3
A08 Complete documentation of required data	1.92 (1.04)	46.2	25.9	19.2	6.8	1.9	27.8
A09 Patient teaching about illness, tests, and diagnostics	1.95 (1.15)	45.9	32.0	6.0	13.2	3.0	22.2
A10 Emotional support for the patient and/or family	1.90 (1.05)	45.9	30.8	12.4	9.0	1.9	23.3
A11 Patient bathing and skin care	2.22 (1.26)	40.2	22.9	16.5	15.0	5.3	36.8
A12 Mouth care	2.34 (1.41)	39.5	25.2	7.5	17.3	10.5	35.3
A13 Hand washing	1.57 (1.08)	72.6	10.9	7.1	6.0	3.4	16.5
A14 Discharge planning and patient education	1.83 (1.18)	59.4	15.0	12.0	10.2	3.4	25.6
A15 Bedside glucose monitoring as ordered	1.33 (0.68)	75.6	18.0	5.3	0.0	1.1	6.4
A16 Patient assessment performed each shift	1.43 (0.83)	72.9	15.8	7.5	2.6	1.1	11.3
A17 Focused reassessment according to patient condition	1.59 (0.86)	59.4	27.1	9.4	3.0	1.1	13.5
A18 IV/central-line site care and assessment	1.39 (0.76)	72.9	18.4	6.4	1.1	1.1	8.6
A19 Response to call light initiated within 5 min	1.63 (0.94)	60.2	24.1	9.8	4.5	1.5	15.8
A20 PRN medication requests addressed within 15 min	1.56 (0.82)	60.2	27.4	9.8	1.5	1.1	12.4
A21 Assessment of medication effectiveness	1.66 (1.01)	59.8	25.6	5.6	6.8	2.3	14.7
A22 Attendance at interdisciplinary care conferences	1.89 (1.09)	49.2	25.2	16.9	4.9	3.8	25.6
A23 Assistance with toileting within 5 min	1.77 (1.01)	51.1	33.5	4.9	8.6	1.9	15.4
A24 Skin and wound care	1.40 (0.70)	69.2	24.1	5.6	0.0	1.1	6.8
A25 Adequate surveillance of confused/cognitively impaired patients	1.41 (0.78)	72.2	18.0	7.1	1.5	1.1	9.8

**Table 3:** Distribution of Responses to Part B: Reasons for Missed Nursing Care (N = 266).

Item/Reason	Mean (SD)	Not a Reason %	Minor %	Moderate %	Significant %	Moderate/Significant %
B01 Inadequate number of staff	3.38 (0.95)	7.9	9.4	19.2	63.5	82.7
B02 Urgent patient situations	3.08 (1.19)	18.0	12.4	13.5	56.0	69.5
B03 Unexpected increase in patient volume and/or acuity	3.41 (0.96)	8.6	7.9	17.3	66.2	83.5
B04 Inadequate assistive and/or clerical personnel	3.41 (0.91)	6.8	8.6	21.1	63.5	84.6
B05 Unbalanced patient assignments	2.91 (1.07)	15.4	15.4	31.6	37.6	69.2
B06 Medications unavailable when needed	2.76 (1.19)	23.3	15.0	23.7	38.0	61.7
B07 Inadequate handoff from the previous shift/sending unit	2.33 (1.13)	30.5	28.2	18.8	22.6	41.4
B08 Other departments did not provide needed care	2.65 (1.18)	24.4	18.8	23.7	33.1	56.8
B09 Supplies/equipment unavailable when needed	2.74 (1.18)	21.8	20.3	20.3	37.6	57.9
B10 Supplies/equipment not functioning properly	2.70 (1.18)	21.8	22.9	18.4	36.8	55.3
B11 Lack of backup support from team members	2.50 (1.18)	28.9	20.7	22.2	28.2	50.4
B12 Communication problems with ancillary/support departments	2.56 (1.17)	26.3	21.4	22.6	29.7	52.3
B13 Communication problems within the nursing team	2.34 (1.16)	32.0	25.6	18.8	23.7	42.5
B14 Communication problems with medical staff	2.41 (1.16)	29.3	26.3	18.8	25.6	44.4
B15 Nursing assistant did not report that care was not provided	2.46 (1.16)	26.7	27.8	18.0	27.4	45.5
B16 Caregiver off the unit or unavailable	2.53 (1.17)	27.1	21.4	22.6	28.9	51.5
B17 Heavy admission and discharge activity	3.08 (1.07)	12.8	15.4	22.9	48.9	71.8
B18 Emotional or physical exhaustion	3.30 (0.98)	7.5	15.0	17.3	60.2	77.4
B19 Inadequate supervision of nursing assistants	2.66 (1.15)	22.6	21.1	24.4	32.0	56.4
B20 Interruptions and simultaneous tasks	3.23 (1.06)	12.0	10.9	19.5	57.5	77.1
B21 Lack of cues or reminders	2.58 (1.09)	21.1	25.2	28.2	25.6	53.8
B22 Inadequate leadership support	2.81 (1.18)	21.8	15.0	23.3	39.8	63.2

**Table 4:** Exploratory factor analysis of Part B items (N = 266).

Item/Reason	Pattern F1	Pattern F2	Structure F1	Structure F2	h^2^	u^2^	Primary Factor
B01 Inadequate number of staff	−0.356	1.093	0.363	0.859	0.810	0.190	F2
B02 Urgent patient situations	−0.007	0.728	0.472	0.723	0.523	0.477	F2
B03 Unexpected increase in patient volume and/or acuity	−0.099	0.972	0.541	0.907	0.829	0.171	F2
B04 Inadequate assistive and/or clerical personnel	0.017	0.852	0.577	0.863	0.745	0.255	F2
B05 Unbalanced patient assignments	0.239	0.591	0.627	0.748	0.591	0.409	F2
B06 Medications unavailable when needed	0.664	0.061	0.704	0.498	0.498	0.502	F1
B07 Inadequate handoff from the previous shift/sending unit	0.811	−0.091	0.752	0.443	0.569	0.431	F1
B08 Other departments did not provide needed care	0.713	0.026	0.730	0.495	0.534	0.466	F1
B09 Supplies/equipment unavailable when needed	0.884	−0.029	0.865	0.552	0.749	0.251	F1
B10 Supplies/equipment not functioning properly	0.962	−0.116	0.886	0.517	0.792	0.208	F1
B11 Lack of backup support from team members	0.854	−0.005	0.851	0.557	0.724	0.276	F1
B12 Communication problems with ancillary/support departments	0.790	0.122	0.870	0.641	0.765	0.235	F1
B13 Communication problems within the nursing team	0.948	−0.093	0.887	0.531	0.791	0.209	F1
B14 Communication problems with medical staff	0.872	−0.002	0.871	0.571	0.758	0.242	F1
B15 Nursing assistant did not report that care was not provided	0.895	−0.068	0.850	0.521	0.726	0.274	F1
B16 Caregiver off the unit or unavailable	0.874	−0.039	0.848	0.536	0.720	0.280	F1
B17 Heavy admission and discharge activity	0.195	0.683	0.644	0.811	0.680	0.320	F2
B18 Emotional or physical exhaustion	0.280	0.678	0.726	0.862	0.788	0.212	F2
B19 Inadequate supervision of nursing assistants	0.732	0.136	0.822	0.618	0.686	0.314	F1
B20 Interruptions and simultaneous tasks	0.281	0.613	0.684	0.798	0.682	0.318	F2
B21 Lack of cues or reminders	0.624	0.261	0.796	0.672	0.673	0.327	F1
B22 Inadequate leadership support	0.716	0.150	0.815	0.621	0.677	0.323	F1

Note: Because promax rotation produces correlated factors, pattern coefficients are standardized regression coefficients and are not restricted to the interval −1 to 1. For B01, the pattern coefficient of 1.093 on Factor 2 occurred together with a negative pattern coefficient on Factor 1 (−0.356). The corresponding structure coefficient (0.859), communality (0.810), and positive uniqueness (0.190) do not indicate a Heywood case.

**Table 5:** Internal Consistency of the Retained Part B Factors.

Factor	Items	Ordinal Alpha (95% CI)	McDonald’s Omega (95% CI)
F1: Organizational resources, coordination, and support	14	0.967 (0.955–0.976)	0.968 (0.956–0.976)
F2: Staffing and workload pressure	8	0.943 (0.917–0.961)	0.943 (0.919–0.961)

**Table 6:** Sensitivity Analysis Based on Unique Exact Response Patterns.

Outcome	Primary Analysis	Sensitivity Analysis/Comparison
Analytical sample	N = 266 responses	n = 200 unique exact response patterns
Part A threshold proportions	All responses	Maximum absolute difference: 3.5 percentage points
Part B threshold proportions	All responses	Maximum absolute difference: 3.6 percentage points
Part A item ranking	Reference ranking	Spearman ρ = 0.993
Part B item ranking	Reference ranking	Spearman ρ = 0.995
Factor retention	Two factors	Two factors
Factor congruence	Reference solution	F1 = 0.995; F2 = 0.990
RMSR	0.061	0.062

## Data Availability

The raw data supporting the conclusions of this article was not made publicly available due to the risk of indirect participant identification, as the combination of clinical department answers and demographic characteristics (e.g., age group) could allow individual respondents to be identified. Confidentiality was explicitly guaranteed to the participants. The raw dataset can be made available by the corresponding author on reasonable request.

## Supplementary Materials

The following supporting information can be downloaded at: https://www.mdpi.com/article/10.3390/healthcare14183122/s1, Supplementary File: MISSCARE Survey Translated to Romanian.

## Abbreviations

The following abbreviations are used in this manuscript:

CFA	Confirmatory Factor Analysis
CHERRIES	Checklist for Reporting Results of Internet E-Surveys
CI	Confidence Interval
EFA	Exploratory Factor Analysis
IV	Intravenous
MNC	Missed Nursing Care
MISSCARE	Missed Nursing Care Survey
SD	Standard Deviation
F1	Factor 1: Organizational resources, coordination, and support
F2	Factor 2: Staffing and workload pressure
