# Supplementary material for: Cross-Cultural Adaptation and Preliminary Psychometric Evaluation of the Romanian Version of the Revised MISSCARE Survey: A Single-Centre Pilot Study
Source: Healthcare (Basel). 2026 Sep 21;14(18):3122. doi: 10.3390/healthcare14183122 (PMC13606467; doi:10.3390/healthcare14183122)
Supplement: Supplementary file 1 [file healthcare-14-03122-s001.zip › healthcare-4540129-supplementary.pdf]

ÎNGRIJIRI DE NURSING OMISE (*Chestionarul MISSCARE*)
Beatrice J. Kalisch

1. **Denumirea secției** în care lucrați: ______________________________

2. Îmi petrec **cea mai mare parte a timpului meu de lucru** în această secție: ______ da ______ nu

3. **Cel mai înalt nivel de educație:**
   1) ______ Școală generală
   2) ______ Absolvent de liceu (sau echivalent)
   3) ______ Absolvent de studii superioare de scurtă durată
   4) ______ Absolvent de studii universitare de licență
   5) ______ Absolvent de studii postuniversitare (master/doctorat)

4. **Dacă sunteți asistent(ă) medical(ă), care este cea mai înaltă calificare:**
   1) ______ Diplomă de asistent medical practician autorizat
   2) ______ Diplomă de asistent medical generalist
   3) ______ Studii superioare de scurtă durată în nursing
   4) ______ Studii universitare de licență în nursing
   5) ______ Studii universitare de licență **în afara** domeniului nursing
   6) ______ Master sau studii superioare în nursing
   7) ______ Master sau studii superioare **în afara** domeniului nursing

5. **Sex:** ______ Feminin ______ Masculin

6. **Vârsta:**
   1) ______ Sub 25 de ani (<25)
   2) ______ 25 - 34 de ani (25-34)
   3) ______ 35 - 44 de ani (35-44)
   4) ______ 45 - 54 de ani (45-54)
   5) ______ 55 - 64 de ani (55-64)
   6) ______ Peste 65 de ani (65+)

7. **Funcția/Rolul:**
   1) ______ Asistent medical generalist
   2) ______ Asistent medical practician autorizat
   3) ______ Infirmier(ă)/personal auxiliar de îngrijire (de ex., îngrijitori/tehnicieni)
   4) ______ Asistent medical șef, asistent medical șef adjunct (de ex., personal de conducere al secției)
   5) ______ Altele [Vă rugăm precizați: ______________________________]

@ Toate drepturile rezervate. Beatrice Kalisch
Vă rugăm să nu reproduceți fără permisiunea autoarei. Revizuit la 9 ianuarie 2019

8. Numărul de **ore lucrate în mod obișnuit pe săptămână** (bifați o singură variantă)
   1) ______ mai puțin de 30 de ore pe săptămână
   2) ______ 30 de ore sau mai mult pe săptămână

9. **Programul de lucru** (bifați varianta care descrie cel mai bine programul dumneavoastră de lucru)
   1) ______ Tura de zi (tură de 8 sau 12 ore)
   2) ______ Tura de după-amiază (tură de 8 sau 12 ore)
   3) ______ Tura de noapte (tură de 8 sau 12 ore)
   4) ______ Rotație între turele de zi, de noapte și de după-amiază

10. **Experiența în funcția actuală:**
   1) ______ Până la 6 luni
   2) ______ Peste 6 luni până la 2 ani
   3) ______ Peste 2 ani până la 5 ani
   4) ______ Peste 5 ani până la 10 ani
   5) ______ Peste 10 ani

11. **Experiența** în **secția actuală de îngrijire a pacienților:**
   1) ______ Până la 6 luni
   2) ______ Peste 6 luni până la 2 ani
   3) ______ Peste 2 ani până la 5 ani
   4) ______ Peste 5 ani până la 10 ani
   5) ______ Peste 10 ani

12. În ce **tură** lucrați cel mai frecvent?
   1) ______ Tură de 8 ore
   2) ______ Tură de 10 ore
   3) ______ Tură de 12 ore
   4) ______ Ture rotative de 8 și 12 ore
   5) ______ Altele [Vă rugăm precizați: ______________________________]

13. În ultimele 3 luni, câte ore **suplimentare** ați lucrat?
   1) _____ Niciuna
   2) _____ 1-12 ore
   3) _____ Peste 12 ore

14. În ultimele 3 luni, câte zile sau ture ați **absentat de la lucru** din cauza bolii, accidentării, nevoii de odihnă suplimentară etc. (excluzând zilele libere aprobate)?
   1) _____ Niciuna

@ Toate drepturile rezervate. Beatrice Kalisch
Vă rugăm să nu reproduceți fără permisiunea autoarei. Revizuit la 9 ianuarie 2019

MINISTERUL JUSTIȚIEI Aut. nr. 29441/2010 PREDOȘANU VASILE Engleză TRADUCĂTOR

2) _____ 1 zi sau tură
3) _____ 2-3 zile sau ture
4) _____ 4-6 zile sau ture
5) _____ peste 6 zile sau ture

15. Intenționați să **părăsiți postul actual?**
 1) _____ în următoarele 6 luni
 2) _____ în următorul an
 3) _____ nu intenționez să plec

16. Cât de des considerați că **personalul secției este suficient?**
 1) ______ 100% din timp
 2) ______ 75% din timp
 3) ______ 50% din timp
 4) ______ 25% din timp
 5) ______ 0% din timp

17. **În tura actuală sau în ultima tură** în care ați lucrat, de câți **pacienți** v-ați ocupat?

____

17-a. câte **internări de pacienți** ați avut (inclusiv transferurile în secție)?

____

17-b. câte **externări de pacienți** ați avut (inclusiv transferurile din secție)?

____

Vă rugăm să bifați un singur **răspuns pentru fiecare întrebare.**

	Foarte satisfăcut(ă)	Satisfăcut(ă)	Neutru	Nesatisfăcut (ă)	Foarte nesatisfăcut (ă)
18. **Cât de satisfăcut(ă)** sunteți de **postul dumneavoastră actual**?					
19. Independent de postul actual, **cât de satisfăcut(ă)** sunteți de faptul că sunteți **asistent(ă) medical(ă) sau infirmier(ă)?**					

@ Toate drepturile rezervate. Beatrice Kalisch
Vă rugăm să nu reproduceți fără permisiunea autoarei. Revizuit la 9 ianuarie 2019

20. **Cât de satisfăcut(ă)** sunteți de **nivelul muncii în echipă din această secție?**					

Secțiunea A — Îngrijiri de nursing omise

Asistenții medicali se confruntă frecvent cu solicitări multiple asupra timpului lor, ceea ce îi obligă să își restabilească prioritățile și să nu realizeze toate îngrijirile de care au nevoie pacienții lor. După cunoștințele dumneavoastră, **cât de frecvent** sunt următoarele elemente ale **îngrijirilor de nursing OMISE de către personalul de nursing (inclusiv dumneavoastră) din secția dumneavoastră?** ***Bifați o singură căsuță pentru fiecare element.***

	**Întotdeauna** omisă	**Frecvent** omisă	**Ocazional** omisă	**Rar** omisă	**Niciodată** omisă
**1) Mobilizarea/deplasarea pacientului de trei ori pe zi sau conform indicațiilor**					
**2) Schimbarea poziției pacientului la fiecare 2 ore**					
**3) Alimentarea pacientului cât timp mâncarea este încă caldă**					
**4) Pregătirea mesei pentru pacientul care se alimentează singur**					
**5) Administrarea medicației în interval de 30 de minute înainte sau după ora programată**					
**6) Măsurarea funcțiilor vitale conform indicațiilor**					
**7) Monitorizarea bilanțului hidric (aport/eliminare)**					
**8) Documentarea completă a tuturor datelor necesare**					
**9) Educarea pacientului privind boala, analizele și investigațiile diagnostice**					
**10) Suport emoțional acordat pacientului și/sau familiei**					
**11) Îmbăierea pacientului/îngrijirea tegumentelor**					
**12) Igiena cavității bucale**					

@ Toate drepturile rezervate. Beatrice Kalisch
Vă rugăm să nu reproduceți fără permisiunea autoarei. Revizuit la 9 ianuarie 2019

MINISTERUL JUSTIȚIEI Autor. 29441/2010 PREDOSANU VASILE Engleză TRADUCĂTOR AUTORIZAT

13) Spălarea mâinilor					
14) Planificarea externării și educarea pacientului la externare					
15) Monitorizarea glicemiei la patul pacientului conform indicațiilor					
16) Evaluarea pacientului efectuată în fiecare tură					

	Întotdeauna omisă	Frecvent omisă	Ocazional omisă	Rar omisă	Niciodată omisă
17) Reevaluări țintite în funcție de starea pacientului					
18) Îngrijirea și evaluarea locului de inserție a cateterului venos periferic/central conform protocolului spitalului					
19) Răspunsul la sistemul de apel al pacientului inițiat în maximum 5 minute					
20) Solicitările de medicație la nevoie rezolvate în maximum 15 minute					
21) Evaluarea eficacității medicației					
22) Participarea la ședințele interdisciplinare de îngrijire ori de câte ori au loc					
23) Asistarea la nevoile de eliminare în maximum 5 minute de la solicitare					
24) Îngrijirea tegumentelor/plăgilor					
25) Supravegherea adecvată a pacienților confuzi/cu deficite cognitive					

Secțiunea B—Motivele omiterii îngrijirilor de nursing

Gândindu-vă la îngrijirile de nursing omise în secția dumneavoastră de către întregul personal (așa cum ați indicat în Partea 1 a acestui chestionar), indicați **MOTIVELE pentru care îngrijirile de nursing sunt OMISE** în secția dumneavoastră. ***Bifați o singură căsuță pentru fiecare element.***

@ Toate drepturile rezervate. Beatrice Kalisch
Vă rugăm să nu reproduceți fără permisiunea autoarei. Revizuit la 9 ianuarie 2019

MINISTERUL JUSTIȚIEI Aut. nr. 28441/2010 PREDOȘANU VASILE

	Motiv semnificativ	Motiv moderat	Motiv minor	NU este un motiv pentru îngrijirile omise
**1) Număr insuficient de personal**				
**2) Situații urgente ale pacienților (de ex., agravarea stării unui pacient)**				
**3) Creștere neașteptată a numărului de pacienți și/sau a gravității cazurilor din secție**				
**4) Număr insuficient de personal auxiliar și/sau administrativ (de ex., infirmieri, tehnicieni, registratori medicali etc.)**				
**5) Repartizare dezechilibrată a pacienților**				

	Motiv semnificativ	Motiv moderat	Motiv minor	NU este un motiv pentru îngrijirile omise
**6) Medicamentele nu au fost disponibile atunci când a fost nevoie**				
**7) Predare inadecvată a turei anterioare sau de la secția care a transferat pacientul**				
**8) Alte departamente nu au furnizat îngrijirile necesare (de ex., kinetoterapia nu a efectuat mobilizarea)**				
**9) Materialele/echipamentele nu au fost disponibile atunci când a fost nevoie**				
**10) Materialele/echipamentele nu au funcționat corespunzător atunci când a fost nevoie**				
**11) Lipsa sprijinului din partea membrilor echipei**				
**12) Tensiuni sau deficiențe de comunicare cu alte DEPARTAMENTE AUXILIARE/DE SUPORT**				
**13) Tensiuni sau deficiențe de comunicare în cadrul ECHIPEI DE NURSING**				
**14) Tensiuni sau deficiențe de comunicare cu PERSONALUL MEDICAL**				

@ Toate drepturile rezervate. Beatrice Kalisch
Vă rugăm să nu reproduceți fără permisiunea autoarei. Revizuit la 9 ianuarie 2019

MINISTERUL JUSTIȚIEI Aut. nr. 29441/2010 PREDOSANU VASILE Engleză TRADUCĂTOR

**15) Infirmierul nu a comunicat că îngrijirea nu a fost efectuată**				
**16) Îngrijitorul a fost plecat din secție sau indisponibil**				
**17) Activitate intensă de internări și externări**				
**18) Epuizare emoțională sau fizică**				
**19) Supervizare inadecvată a infirmierilor**				
**20) Întreruperi/Sarcini multiple simultane**				
**21) Lipsa unor indicii/memento-uri**				
**22) Sprijin inadecvat din partea conducerii**				

VĂ MULȚUMIM PENTRU PARTICIPARE!

Subsemnatul **PREDOŞANU VASILE**, traducător autorizat, cu autorizația nr. 29441/2010, eliberată de Ministerul Justiției, certific exactitatea traducerii efectuate din limba ENGLEZĂ în limba ROMÂNĂ, că textul prezentat a fost tradus complet, fără omisiuni, și că, prin traducere, înscrisului nu i-a fost denaturat conținutul și sensul.

PREDOŞANU VASILE

Traducător autorizat de limba engleză

MINISTERUL JUSTIȚIEI Aut. nr. 29441/2010 PREDOŞANU VASILE Engleză TRADUCĂTOR AUTORIZAT

@ Toate drepturile rezervate. Beatrice Kalisch
Vă rugăm să nu reproduceți fără permisiunea autoarei. Revizuit la 9 ianuarie 2019

ÎNGRIJIRI DE NURSING OMISE (*Chestionarul MISSCARE*)
Beatrice J. Kalisch

1. **Denumirea secției** în care lucrați: ______________________________

2. Îmi petrec **cea mai mare parte a timpului meu de lucru** în această secție: ______ da ______ nu

3. **Cel mai înalt nivel de educație:**
   1) ______ Școală generală
   2) ______ Absolvent de liceu (sau echivalent)
   3) ______ Absolvent de studii superioare de scurtă durată
   4) ______ Absolvent de studii universitare de licență
   5) ______ Absolvent de studii postuniversitare (master/doctorat)

4. **Dacă sunteți asistent(ă) medical(ă), care este cea mai înaltă calificare:**
   1) ______ Diplomă de asistent medical practician autorizat
   2) ______ Diplomă de asistent medical generalist
   3) ______ Studii superioare de scurtă durată în nursing
   4) ______ Studii universitare de licență în nursing
   5) ______ Studii universitare de licență **în afara** domeniului nursing
   6) ______ Master sau studii superioare în nursing
   7) ______ Master sau studii superioare **în afara** domeniului nursing

5. **Sex:** ______ Feminin ______ Masculin

6. **Vârsta:**
   1) ______ Sub 25 de ani (<25)
   2) ______ 25 - 34 de ani (25-34)
   3) ______ 35 - 44 de ani (35-44)
   4) ______ 45 - 54 de ani (45-54)
   5) ______ 55 - 64 de ani (55-64)
   6) ______ Peste 65 de ani (65+)

7. **Funcția/Rolul:**
   1) ______ Asistent medical generalist
   2) ______ Asistent medical practician autorizat
   3) ______ Infirmier(ă)/personal auxiliar de îngrijire (de ex., îngrijitori/tehnicieni)
   4) ______ Asistent medical șef, asistent medical șef adjunct (de ex., personal de conducere al secției)
   5) ______ Altele [Vă rugăm precizați: ______________________________]

@ Toate drepturile rezervate. Beatrice Kalisch
Vă rugăm să nu reproduceți fără permisiunea autoarei. Revizuit la 9 ianuarie 2019

MINISTERUL JUSTIȚIEI Aut. nr. 29441/2010 PREDOȘANU VASILE Engleză TRADUCĂTOR

8. Numărul de **ore lucrate în mod obișnuit pe săptămână** (bifați o singură variantă)
   1) ______ mai puțin de 30 de ore pe săptămână
   2) ______ 30 de ore sau mai mult pe săptămână

9. **Programul de lucru** (bifați varianta care descrie cel mai bine programul dumneavoastră de lucru)
   1) ______ Tura de zi (tură de 8 sau 12 ore)
   2) ______ Tura de după-amiază (tură de 8 sau 12 ore)
   3) ______ Tura de noapte (tură de 8 sau 12 ore)
   4) ______ Rotație între turele de zi, de noapte și de după-amiază

10. **Experiența în funcția actuală:**
   1) ______ Până la 6 luni
   2) ______ Peste 6 luni până la 2 ani
   3) ______ Peste 2 ani până la 5 ani
   4) ______ Peste 5 ani până la 10 ani
   5) ______ Peste 10 ani

11. **Experiența** în **secția actuală de îngrijire a pacienților:**
   1) ______ Până la 6 luni
   2) ______ Peste 6 luni până la 2 ani
   3) ______ Peste 2 ani până la 5 ani
   4) ______ Peste 5 ani până la 10 ani
   5) ______ Peste 10 ani

12. În ce **tură** lucrați cel mai frecvent?
   1) ______ Tură de 8 ore
   2) ______ Tură de 10 ore
   3) ______ Tură de 12 ore
   4) ______ Ture rotative de 8 și 12 ore
   5) ______ Altele [Vă rugăm precizați: ______________________________]

13. În ultimele 3 luni, câte ore **suplimentare** ați lucrat?
   1) _____ Niciuna
   2) _____ 1-12 ore
   3) _____ Peste 12 ore

14. În ultimele 3 luni, câte zile sau ture ați **absentat de la lucru** din cauza bolii, accidentării, nevoii de odihnă suplimentară etc. (excluzând zilele libere aprobate)?
   1) _____ Niciuna

@ Toate drepturile rezervate. Beatrice Kalisch
Vă rugăm să nu reproduceți fără permisiunea autoarei. Revizuit la 9 ianuarie 2019

MINISTERUL JUSTIȚIEI Aut. nr. 29441/2010 PREDOȘANU VASILE Engleză

2) _____ 1 zi sau tură
3) _____ 2-3 zile sau ture
4) _____ 4-6 zile sau ture
5) _____ peste 6 zile sau ture

15. Intenționați să **părăsiți postul actual?**
   1) _____ în următoarele 6 luni
   2) _____ în următorul an
   3) _____ nu intenționez să plec

16. Cât de des considerați că **personalul secției este suficient?**
   1) ______ 100% din timp
   2) ______ 75% din timp
   3) ______ 50% din timp
   4) ______ 25% din timp
   5) ______ 0% din timp

17. **În tura actuală sau în ultima tură** în care ați lucrat, de câți **pacienți** v-ați ocupat?

____

17-a. câte **internări de pacienți** ați avut (inclusiv transferurile în secție)?

____

17-b. câte **externări de pacienți** ați avut (inclusiv transferurile din secție)?

____

Vă rugăm să bifați un singur **răspuns pentru fiecare întrebare.**

	Foarte satisfăcut(ă)	Satisfăcut(ă)	Neutru	Nesatisfăcut (ă)	Foarte nesatisfăcut (ă)
18. **Cât de satisfăcut(ă)** sunteți de **postul dumneavoastră actual**?					
19. Independent de postul actual, **cât de satisfăcut(ă)** sunteți de faptul că sunteți **asistent(ă) medical(ă) sau infirmier(ă)?**					

@ Toate drepturile rezervate. Beatrice Kalisch
Vă rugăm să nu reproduceți fără permisiunea autoarei. Revizuit la 9 ianuarie 2019

20. **Cât de satisfăcut(ă)** sunteți de **nivelul muncii în echipă din această secție?**					

Secțiunea A — Îngrijiri de nursing omise

Asistenții medicali se confruntă frecvent cu solicitări multiple asupra timpului lor, ceea ce îi obligă să își restabilească prioritățile și să nu realizeze toate îngrijirile de care au nevoie pacienții lor. După cunoștințele dumneavoastră, **cât de frecvent** sunt următoarele elemente ale **îngrijirilor de nursing <u>OMISE</u> de către personalul de nursing (inclusiv dumneavoastră) din secția dumneavoastră?** ***Bifați o singură căsuță pentru fiecare element.***

	**Întotdea una** omisă	**Frecvent** omisă	**Ocazional** omisă	**Rar** omisă	**Niciod ată** omisă
**1) Mobilizarea/deplasarea pacientului de trei ori pe zi sau conform indicațiilor**					
**2) Schimbarea poziției pacientului la fiecare 2 ore**					
**3) Alimentarea pacientului cât timp mâncarea este încă caldă**					
**4) Pregătirea mesei pentru pacientul care se alimentează singur**					
**5) Administrarea medicației în interval de 30 de minute înainte sau după ora programată**					
**6) Măsurarea funcțiilor vitale conform indicațiilor**					
**7) Monitorizarea bilanțului hidric (aport/eliminare)**					
**8) Documentarea completă a tuturor datelor necesare**					
**9) Educarea pacientului privind boala, analizele și investigațiile diagnostice**					
**10) Suport emoțional acordat pacientului și/sau familiei**					
**11) Îmbăierea pacientului/îngrijirea tegumentelor**					
**12) Igiena cavității bucale**					

@ Toate drepturile rezervate. Beatrice Kalisch
Vă rugăm să nu reproduceți fără permisiunea autoarei. Revizuit la 9 ianuarie 2019

MINISTERUL JUSTIȚIEI Autoriz.nr.29441/2010 * PREDOȘANU VASILE * TRADUCĂTOR Engleză

13) Spălarea mâinilor					
14) Planificarea externării și educarea pacientului la externare					
15) Monitorizarea glicemiei la patul pacientului conform indicațiilor					
16) Evaluarea pacientului efectuată în fiecare tură					

	Întotdeauna omisă	Frecvent omisă	Ocazional omisă	Rar omisă	Niciodată omisă
17) Reevaluări țintite în funcție de starea pacientului					
18) Îngrijirea și evaluarea locului de inserție a cateterului venos periferic/central conform protocolului spitalului					
19) Răspunsul la sistemul de apel al pacientului inițiat în maximum 5 minute					
20) Solicitările de medicație la nevoie rezolvate în maximum 15 minute					
21) Evaluarea eficacității medicației					
22) Participarea la ședințele interdisciplinare de îngrijire ori de câte ori au loc					
23) Asistarea la nevoile de eliminare în maximum 5 minute de la solicitare					
24) Îngrijirea tegumentelor/plăgilor					
25) Supravegherea adecvată a pacienților confuzi/cu deficite cognitive					

Secțiunea B—Motivele omiterii îngrijirilor de nursing

Gândindu-vă la îngrijirile de nursing omise în secția dumneavoastră de către întregul personal (așa cum ați indicat în Partea 1 a acestui chestionar), indicați **MOTIVELE pentru care îngrijirile de nursing sunt OMISE** în secția dumneavoastră. ***Bifați o singură căsuță pentru fiecare element.***

@ Toate drepturile rezervate. Beatrice Kalisch
Vă rugăm să nu reproduceți fără permisiunea autoarei. Revizuit la 9 ianuarie 2019

MINISTERUL JUSTIȚIEI
Aut. nr. 29441/2010
PREDOȘANU VASILE
Engleză
TRADUCĂTOR

	Motiv semnificativ	Motiv moderat	Motiv minor	NU este un motiv pentru îngrijirile omise
1) Număr insuficient de personal				
2) Situații urgente ale pacienților (de ex., agravarea stării unui pacient)				
3) Creștere neașteptată a numărului de pacienți și/sau a gravității cazurilor din secție				
4) Număr insuficient de personal auxiliar și/sau administrativ (de ex., infirmieri, tehnicieni, registratori medicali etc.)				
5) Repartizare dezechilibrată a pacienților				

	Motiv semnificativ	Motiv moderat	Motiv minor	NU este un motiv pentru îngrijirile omise
6) Medicamentele nu au fost disponibile atunci când a fost nevoie				
7) Predare inadecvată a turei anterioare sau de la secția care a transferat pacientul				
8) Alte departamente nu au furnizat îngrijirile necesare (de ex., kinetoterapia nu a efectuat mobilizarea)				
9) Materialele/echipamentele nu au fost disponibile atunci când a fost nevoie				
10) Materialele/echipamentele nu au funcționat corespunzător atunci când a fost nevoie				
11) Lipsa sprijinului din partea membrilor echipei				
12) Tensiuni sau deficiențe de comunicare cu alte DEPARTAMENTE AUXILIARE/DE SUPORT				
13) Tensiuni sau deficiențe de comunicare în cadrul ECHIPEI DE NURSING				
14) Tensiuni sau deficiențe de comunicare cu PERSONALUL MEDICAL				

@ Toate drepturile rezervate. Beatrice Kalisch
Vă rugăm să nu reproduceți fără permisiunea autoarei. Revizuit la 9 ianuarie 2019

MINISTERUL JUSTIȚIEI Aut. nr. 29441/2010 PREDOȘANU VASILE Engleză TRADUCĂTOR

**15) Infirmierul nu a comunicat că îngrijirea nu a fost efectuată**				
**16) Îngrijitorul a fost plecat din secție sau indisponibil**				
**17) Activitate intensă de internări și externări**				
**18) Epuizare emoțională sau fizică**				
**19) Supervizare inadecvată a infirmierilor**				
**20) Întreruperi/Sarcini multiple simultane**				
**21) Lipsa unor indicii/memento-uri**				
**22) Sprijin inadecvat din partea conducerii**				

VĂ MULȚUMIM PENTRU PARTICIPARE!

Subsemnatul **PREDOŞANU VASILE**, traducător autorizat, cu autorizația nr. 29441/2010, eliberată de Ministerul Justiției, certific exactitatea traducerii efectuate din limba ENGLEZĂ în limba ROMÂNĂ, că textul prezentat a fost tradus complet, fără omisiuni, și că, prin traducere, înscrisului nu i-a fost denaturat conținutul și sensul.

PREDOŞANU VASILE

Traducător autorizat de limba engleză

@ Toate drepturile rezervate. Beatrice Kalisch
Vă rugăm să nu reproduceți fără permisiunea autoarei. Revizuit la 9 ianuarie 2019
